# 

**Published:** 1886-07

**Authors:** 


### OUR PROSPECTS.

The June number of the Journal completed its first half year, under the new management. The transit from the dullest *old-fogyism* into the full light of modern development and discovery relative to the prevention, treatment and cure of disease, was by no means an inconsiderable or unimportant one. Realizing the fact that nearly all so-called medical journals either from policy or indifference are hedged in by *old school* prejudices and proprieties, it was our belief that these antiquarian notions should give place to others more consistent with the spirit of the times, and what publication so fitting to take the iniative, as the oldest and best known among them, hence the change in its conduct.

We are glad to be able to inform our patrons both old and new, that the change has met with general approval, and is in every way so successful and satisfactory that its future may be considered assured.

*As an advertising medium*, its thirty-three years of circulation among the intelligent of all classes, now largely increasing, makes it second to no other.

A noticable feature of our business department is the filling of orders for any remedy advertised in our columns, or for any obtainable book or publication, at wholesale or publishers rates, and their transmission by mail or otherwise to the place of delivery. That there was a necessity for this feature is shown by the multiplicity of orders received, many of which are from remote localities.

